# Task-Aware Multi-Modal Sensing Control for IoT Systems

**DOI:** 10.3390/s26196001

**Published:** 2026-09-22

**Authors:** Yeunwoong Kyung, Dogyu Han, Jihoon Sung, Geunho Lee, Jeongwook Seo, Haneul Ko, Jaehyuk Lee

**Affiliations:** 1 Department of Electronic Engineering, Seoul National University of Science and Technology, Seoul 01811, Republic of Korea; ywkyung@seoultech.ac.kr (Y.K.); dokyu0201@seoultech.ac.kr (D.H.); 2 Department of Computer Science and Artificial Intelligence, Jeonbuk National University, 567 Baekje-daero, Jeonju-si 54896, Republic of Korea; jh.sung@jbnu.ac.kr; 3 Graduate School of Engineering, University of Miyazaki, Miyazaki 889-2192, Japan; geunho@miyazaki-u.ac.jp; 4 School of Computing and Artificial Intelligence, Hanshin University, Osan 18101, Republic of Korea; jwseo@hs.ac.kr; 5 Department of Electronic Engineering, Kyung Hee University, Yongin 17104, Republic of Korea; 6 Department of Computer Engineering, Seowon University, Cheongju-si 28674, Republic of Korea

**Keywords:** multi-modal sensors, closed-loop framework, IoT system, sensing control, task-aware sensing

## Abstract

Future Internet of Things (IoT) systems increasingly rely on multiple sensing modalities to support sensing-intensive tasks such as localization and smart healthcare. However, always collecting all available sensing data can waste energy and communication resources, whereas aggressive sensing reduction may degrade task-level performance. The key challenge is therefore to adapt sensing operations according to the requirements of the target task while avoiding unnecessary sensing effort. To address this challenge, this article presents Task-Aware Multi-Modal Sensing Control (TA-MSC) for IoT systems. TA-MSC treats sensing operation as a task-level-output-driven closed-loop control problem that identifies a low-cost sensing configuration while satisfying a task-level performance requirement. By analyzing task-level outputs from the inference process, TA-MSC evaluates whether the current sensing configuration is sufficient and updates modality-wise sensing levels accordingly. The selected sensing configuration can be realized through practical control dimensions such as modality selection, sensor activation, channel adjustment, and duty-cycle management. We instantiate TA-MSC through a representative multi-modal localization case study, in which sensing energy is evaluated using literature-based sensing and transmission-energy models rather than direct hardware measurements. In the evaluated cross-device analysis, TA-MSC reduces the mean total modeled sensing energy by 52.8% relative to All-Max, while achieving a Top-1 localization accuracy of 96.14% compared with 97.37% for All-Max and a confidence-satisfaction rate of 98.94% compared with 97.68% for All-Max. These results indicate substantial modeled-energy savings with a modest Top-1 accuracy tradeoff, while the confidence-based task condition is satisfied more frequently.

## 1. Introduction

Future Internet of Things (IoT) systems are evolving from simple data collection platforms into sensing-intensive task processing systems. Instead of merely collecting and uploading raw measurements, IoT systems are increasingly expected to support application-level tasks such as localization, context-aware services, and smart healthcare [1]. In these systems, sensing is not only a passive data acquisition function, but also a system-level operation that directly affects task performance, device energy consumption, communication overhead, and processing cost.

A key characteristic of such applications is their reliance on multiple sensing modalities. For example, localization can exploit heterogeneous information from cellular measurements, Wi-Fi, Bluetooth Low Energy (BLE), inertial sensors, or other device-side sensing sources [1]. These modalities observe different aspects of the surrounding temporal, spatial, and radio environments and can therefore complement each other under diverse operating conditions. At the same time, they differ in energy consumption, measurement availability, and communication overhead. Importantly, the amount of sensing required to support a target task is not necessarily fixed. A rich sensing configuration may be necessary under difficult or ambiguous conditions, whereas a reduced configuration may be sufficient when the inference result is already reliable. As a result, multi-modal sensing provides richer information than a single sensing source, but it also requires careful configuration according to the current task and system conditions.

In practice, many sensing systems still rely on static or predefined sensing policies. Devices may activate a fixed set of sensing modalities or scan a fixed set of channels at predetermined intervals, regardless of the current task condition. Such policies are simple and predictable, but they are often inefficient under dynamic task and system conditions. A fixed configuration may become insufficient when task conditions change, or it may waste resources when the inference result is already reliable. This creates a fundamental sensing-control dilemma: always-on sensing can waste energy and communication resources, whereas aggressive sensing reduction can degrade task-level performance. Therefore, an adaptive sensing system should determine an appropriate sensing configuration according to the current task requirement rather than statically collecting or suppressing sensing data.

Prior studies have addressed sensing and communication overhead through energy-efficient sensing, adaptive sampling, data reduction, and sensor selection [2,3,4,5,6,7]. These techniques provide important mechanisms for reducing sensing cost and adapting sensing operations according to signal, information, energy, or system conditions. More recent studies have further considered application-level information or task-related feedback when adapting sensing operations [8,9,10]. These approaches extend adaptive sensing beyond sensing-side conditions by using model-derived information, such as prediction uncertainty or task-related utility, to guide sensing decisions. In localization, sensing or data acquisition has also been optimized according to localization accuracy, sensor attributes, and energy cost [11,12]. Collectively, these studies demonstrate that adaptive sensing, task-related feedback, and learning-based sensing control have already been investigated in different forms. Therefore, these features alone do not constitute the main distinction of TA-MSC. Instead, TA-MSC explicitly separates the two roles in the sensing-control problem: sensing cost is treated as the optimization objective, whereas task-level sufficiency is imposed as a requirement that the selected sensing configuration must satisfy. This formulation enables heterogeneous sensing levels to be jointly adjusted according to inference outputs without conflating sensing efficiency with task performance. For example, Zhou et al. [11] determine the minimum offline sample capacity corresponding to a given expected localization accuracy, whereas Shurrab et al. [12] combine RL-based localization with active node selection considering sensor attributes. However, these localization-oriented approaches do not directly address runtime joint control of heterogeneous multi-modal sensing levels using task-level outputs of the inference process as an explicit condition for sensing-cost reduction.

To address this issue, this article presents Task-Aware Multi-Modal Sensing Control (TA-MSC) for IoT systems. TA-MSC treats sensing operation as a task-level-output-driven closed-loop control problem. Rather than minimizing sensing cost unconditionally, TA-MSC seeks a low-cost sensing configuration while requiring the selected configuration to satisfy a task-level performance condition. Task-level outputs produced by the inference process, such as confidence, are used as control-relevant feedback to assess whether the current sensing configuration is sufficient and to update modality-wise sensing levels accordingly. The selected configuration can then be translated into platform-supported control actions, such as modality selection, sensor activation, channel adjustment, and duty-cycle management.

A key distinction of TA-MSC is not adaptive sensing, task-related feedback, or closed-loop operation alone, as these concepts have already been explored in prior studies. Rather, TA-MSC structures the sensing-control problem by explicitly separating sensing cost as the optimization objective from task-level sufficiency as a constraint on the selected sensing configuration. Task-level outputs from the inference process are used to evaluate this sufficiency condition, while heterogeneous modality-specific sensing levels are jointly adjusted to reduce sensing cost. In the localization case study, this principle is instantiated by minimizing modeled sensing energy subject to a confidence-gap requirement of the fused localization estimator.

The main contributions of this article are summarized as follows.

- We consider multi-modal sensing as a task-aware control problem that minimizes sensing effort while satisfying an explicit task-level performance requirement.
- We present TA-MSC, which connects task-level outputs from an inference model to the joint configuration of heterogeneous sensing modalities through practical multi-level sensing-control dimensions.
- We demonstrate TA-MSC through an OutFin-based multi-modal localization case study using literature-based sensing and transmission-energy models. TA-MSC reduces the mean modeled sensing energy while maintaining a high confidence-satisfaction rate.

The remainder of this article is organized as follows. Section 2 reviews existing sensing-efficiency and adaptive sensing approaches and positions TA-MSC relative to prior studies. Section 3 presents the TA-MSC architecture and sensing-control framework, and Section 4 presents a representative localization case study. Section 5 discusses open research issues. Finally, Section 6 concludes this article.

## 2. From Sensing Efficiency to Task-Aware Control

This section reviews representative energy-efficient, adaptive, and task-aware sensing approaches and positions TA-MSC relative to prior studies. We first discuss sensing mechanisms driven primarily by sensing-side information and resource conditions, and then review recent approaches that incorporate application-level or task-related information into sensing decisions. We finally discuss localization-oriented sensing optimization and clarify the control perspective addressed by TA-MSC.

### 2.1. Energy-Efficient and Adaptive Sensing

Existing IoT sensing systems have adopted various mechanisms to reduce sensing and communication overhead. Energy-efficient sensing techniques, such as sleep scheduling, duty cycling, and sensor activation control, reduce device energy consumption and extend network lifetime [2,3]. Adaptive sampling and data-reduction techniques decrease the amount of acquired or transmitted data by adjusting sampling rates or data representations according to signal or system conditions [4,5]. Redundancy- and correlation-aware approaches further exploit statistical relationships among sensing streams to suppress duplicate or less informative observations [6,7].

Learning-based approaches have extended these mechanisms by allowing sensing policies to adapt to changing sensing and system conditions. For example, learning-based sensor selection can reduce sensing energy by selecting an active sensor subset according to inter-sensor correlation, sensing cost, and available energy, while estimating measurements from inactive sensors [13].

These approaches provide important mechanisms for controlling sensing effort and form practical building blocks for adaptive IoT sensing. However, their control criteria are primarily derived from sensing-side characteristics, information value, or resource conditions. As sensing systems increasingly support downstream inference tasks, sensing decisions can further benefit from information directly associated with the target task.

### 2.2. Task-Aware and Localization-Oriented Sensing

More recent studies have incorporated application-level or model-derived information into sensing decisions. Uncertainty-aware autonomous sensing uses prediction uncertainty to concentrate sensing resources on informative measurements while reducing unnecessary observations [8]. In multi-modal perception, cross-modality adaptive sensing approaches use task-related information to determine whether additional sensing modalities or higher sensing rates are beneficial [9]. Other recent frameworks further estimate modality-specific task information from a perception model and adapt physical sensing parameters, such as sampling frequency, resolution, or sensing range, during operation [10]. These studies demonstrate that task-related feedback and closed-loop sensing adaptation have already been explored in different application domains.

Related sensing-optimization approaches have also been investigated for localization. Zhou et al. [11] study energy-efficient fingerprint database construction and derive the minimum sample capacity corresponding to a given expected localization accuracy using an information-theoretic model. Their approach therefore connects localization-performance requirements with data-acquisition reduction, but primarily targets offline fingerprint database construction. Shurrab et al. [12] combine Q-learning-based target localization with active sensor selection. Their framework selects sensing nodes according to attributes such as activation cost, residual energy, measurement confidence, and distance, while the RL agent guides the localization process toward the target region.

Collectively, these studies show that task-related information can be incorporated into sensing decisions in multiple ways, ranging from uncertainty-aware measurement timing and multi-modal adaptation to localization-oriented data or sensor selection. Thus, adaptive sensing, task feedback, and learning-based sensing control should not individually be regarded as unique features of a task-aware sensing framework.

Table 1 summarizes representative adaptive, task-aware, and localization-oriented sensing approaches according to their application domain, use of multi-modal inference, decision basis, sensing-control dimensions, and treatment of task performance.

### 2.3. Positioning of TA-MSC

TA-MSC builds on these prior directions but focuses on a different control perspective. Rather than treating adaptive sensing or task-aware feedback itself as the main contribution, TA-MSC organizes heterogeneous multi-modal sensing control around two explicit elements: sensing cost and a task-level performance requirement. The task-level requirement is evaluated using outputs exposed by the inference process, and the resulting feedback is used to determine whether a lower-cost sensing configuration remains sufficient for the target task.

This perspective differs from localization approaches that primarily optimize offline data acquisition or select sensing nodes according to sensor-side attributes. It also differs from task-aware sensing approaches that incorporate task utility, uncertainty, or modality importance directly into the sensing policy or optimization objective. At the framework level, TA-MSC separates sensing cost as the optimization objective from task-level sufficiency as an explicit constraint: the controller seeks to reduce sensing cost while the selected configuration is required to satisfy the specified task condition.

Accordingly, existing sensing-efficiency mechanisms are not replaced by TA-MSC. Instead, they are interpreted as practical sensing-control dimensions within a task-level-output-driven closed loop. Depending on platform capabilities, the controller can jointly configure modality selection, sensor activation, channel adjustment, and duty-cycle management. In the localization case study presented in Section 4, these principles are instantiated through joint multi-level control of Wi-Fi, BLE, cellular, and onboard sensing, where modeled sensing energy is minimized subject to a confidence-gap requirement of the localization estimator.

## 3. Task-Aware Multi-Modal Sensing Control Framework

This section presents the TA-MSC framework. The objective of TA-MSC is to identify a sensing configuration that satisfies a task-level performance requirement while avoiding unnecessary sensing effort under dynamic task, device, network, and environmental conditions.

TA-MSC is guided by three design rationales. First, sensing-control decisions should use task-level outputs together with sensing cost, rather than being driven by sensing cost alone. Second, the required sensing configuration should be determined according to whether the task-level performance requirement is satisfied under the current operating condition, rather than treating sensing requirements as fixed across operating conditions. Third, sensing effort should be reduced only when the required task condition can be maintained through platform-supported control actions.

The following subsections describe the overall architecture, task-level output interface, task-aware sensing controller, and closed-loop operation of TA-MSC.

### 3.1. Overall Architecture

As illustrated in Figure 1, TA-MSC consists of four major functional components: multi-modal sensing sources, the network, the task processor, and the task-aware sensing controller. Figure 1 illustrates two logical flows among these components. In the forward sensing and inference flow, heterogeneous sensing sources generate observations that are delivered through the network to the task processor, which performs the target inference task and produces task-level outputs. In the feedback control flow, these task-level outputs are provided to the task-aware sensing controller, which assesses task sufficiency and determines the sensing configuration for the next control interval. The resulting control decisions are then applied to the sensing sources, thereby forming a closed sensing–inference–control loop.

*(1) Multi-Modal Sensing Sources:* Multi-modal sensing sources provide heterogeneous observations for the target task, such as wireless and RF measurements, inertial data, environmental measurements, or other domain-specific sensing data. Each modality has different characteristics in terms of measurement availability, energy consumption, and communication overhead. In TA-MSC, these modalities are treated as configurable resources rather than fixed data sources. Depending on the sensing platform or capability, each modality can be activated or deactivated, operated at different sensing levels, scanned with different channel settings, or controlled through different duty-cycle patterns. The sensing sources illustrated in Figure 1 are representative examples; TA-MSC is not restricted to these specific devices or modalities and can incorporate other domain-specific sensing sources whose sensing operations can be configured by the platform.

*(2) Network Layer:* The network layer delivers sensing data from devices to the task processor. Although TA-MSC is not primarily designed as a communication optimization framework, network-related conditions may influence sensing-control decisions. For example, activating additional modalities can increase data volume and transmission energy. Therefore, network conditions can be treated as part of the system context of TA-MSC. Similarly, the communication technologies shown in Figure 1 are illustrative, and TA-MSC does not assume a specific communication protocol or access technology; different wired or wireless communication methods can be used to deliver sensing data and control information depending on the deployment environment.

*(3) Task Processor:* The task processor performs the target inference task using the sensing data collected from the active modalities. In TA-MSC, the task processor produces not only the task result but also task-level outputs that can support sensing control. Rather than assuming a fixed output type, TA-MSC requires an indicator that reflects whether the current sensing configuration sufficiently supports the target task. These task-level outputs provide the control-relevant information used to assess task sufficiency and update the sensing configuration.

*(4) Task-Aware Sensing Controller:* The task-aware sensing controller is the core component of TA-MSC. It receives task-level outputs from the task processor and determines the next sensing configuration. As shown in Figure 1, the controller consists of four logical functions: task-level output analysis, task-sufficiency assessment, control policy, and sensing-control dimensions. These functions connect task-level outputs to practical sensing-control actions such as modality selection, sensor activation, channel adjustment, and duty-cycle management.

### 3.2. Task-Level Outputs and Control Interface

Task-level outputs provide the control-relevant information that connects the task processor and the task-aware sensing controller. They represent how well the current sensing configuration supports the target task. Depending on the application and deployment condition, these outputs may include direct performance indicators during offline evaluation or surrogate indicators produced by the inference process during online operation. TA-MSC does not require a universal output metric; instead, it uses task-level indicators that can reflect whether the current sensing configuration is sufficient for the target task.

In TA-MSC, task-level outputs are not treated only as evaluation metrics. Instead, they serve as the control interface between the task processor and the sensing controller. These outputs indicate whether the current sensing configuration satisfies the required task condition and provide information for assessing whether the sensing configuration should be maintained, reduced, or enriched. Therefore, the task processor should expose control-relevant task-level outputs in a form that can be interpreted by the sensing controller, while the controller maps these outputs to sensing-configuration decisions according to the task requirement and sensing cost.

### 3.3. Task-Aware Sensing Controller

This subsection details the controller-side functions of TA-MSC. Consistent with Figure 1, the controller consists of four logical functions. Since the task-level output interface is discussed in Section 3.2, this subsection focuses on how the controller assesses task sufficiency, maps task-related information and sensing cost into control decisions, and executes the selected decisions through practical sensing-control dimensions.

*Task-sufficiency assessment:* Given the task-level outputs provided through the control interface, the controller assesses whether the current sensing configuration sufficiently supports the target task. The assessment is performed relative to a task-specific performance requirement rather than by assuming that a fixed amount of sensing is always necessary.

When the task-level output indicates that the required task condition is already satisfied, the controller may consider a lower-cost sensing configuration by reducing sensing levels, deactivating selected sensing sources, or increasing inactive periods. In contrast, when the task-level condition is not satisfied, a richer sensing configuration may be required. The assessment therefore provides the information needed to distinguish between sufficient and insufficient sensing configurations under the current task and system context.

In practice, task sufficiency may not be directly observable for every candidate sensing configuration before it is selected. Therefore, the controller may assess or predict task-level performance using current task-level outputs, historical observations, sensing actions, system context, or learned models. The specific assessment method depends on the target task, available task-level outputs, and deployment constraints.

*Control policy:* The control policy maps task-sufficiency assessment and sensing cost into sensing-control decisions. Its objective is to select a sensing configuration that satisfies the task-level requirement while reducing unnecessary sensing effort. This framework-level objective can be generally represented as

(1) $$c^{★}=\arg\min_{c\in C}J_{c}\left(x\right),\;s.t.\;{\hat{Q}}_{c}\left(x\right)\geq \Gamma ,$$

where *c* denotes a multi-modal sensing configuration, C is the set of feasible configurations supported by the sensing platform, and *x* denotes the current system and task context. $J_{c}\left(x\right)$ represents the sensing cost, such as energy consumption or communication overhead. ${\hat{Q}}_{c}\left(x\right)$ denotes the estimated task-level performance indicator for configuration *c* under context *x*. The threshold Γ denotes the required task-level condition, which can be instantiated according to the target task and the selected task-level performance indicator. When a task-level indicator is defined such that a lower value is better, an equivalent transformed indicator or inequality can be used.

This formulation should be interpreted as a framework-level control objective rather than a restriction to a particular optimization algorithm. In online operation, the controller does not necessarily observe the task-level output of every feasible configuration before making a decision. For a candidate configuration that has not yet been applied, ${\hat{Q}}_{c}\left(x\right)$ represents the expected task-level performance under that configuration rather than a directly observed output. It can be estimated using current task-level outputs, historical observations, system context, or learning-based prediction. The specific estimation mechanism depends on the sensing application and the information available to the controller. For example, in an offline evaluation where sufficiently rich multi-modal measurements are already available, candidate sensing configurations can be emulated by retaining only the measurements corresponding to each configuration and evaluating the resulting task output. Regardless of the estimation method, the role of the control policy is to identify a low-cost sensing configuration that is expected to satisfy the required task condition. In this way, the control policy aims to avoid both unnecessary over-sensing and task-performance degradation caused by insufficient sensing.

*Sensing-control dimensions:* The output of the control policy should be translated into practical sensing actions supported by devices and sensing platforms. Representative sensing-control dimensions include modality selection, sensor activation, channel adjustment, and duty-cycle management.

Modality selection determines which sensing modalities are used for the target task. Sensor activation controls whether a sensing source is turned on or off. This is useful when a reduced sensing configuration remains sufficient for the required task condition or when device energy should be conserved. Channel adjustment controls the richness or granularity of sensing within an active modality. In wireless sensing, this may correspond to selecting a subset of channels. Duty-cycle management controls the active and inactive periods of sensing operations. For example, in BLE-based sensing, the controller may adjust the scan window to determine how frequently and how long the scanning is performed.

These control dimensions are not mutually exclusive. A selected sensing configuration may combine multiple dimensions, such as activating selected modalities, scanning only selected channels, reducing the duty cycle, or deactivating a subset of sensors. These dimensions can be combined depending on device capability and sensing interfaces, while the resulting configuration should be selected according to both sensing cost and the required task condition.

### 3.4. Closed-Loop Operation

TA-MSC operates as an iterative sensing-control process. At each control interval, the system applies the selected sensing configuration, performs the target inference task, analyzes task-level outputs, assesses whether the current sensing configuration satisfies the required task condition, and updates the sensing configuration for the next interval. Through this loop, sensing effort can be reduced when a lower-cost configuration is expected to remain sufficient for the target task, whereas richer sensing configurations can be selected when the required task condition is not satisfied.

## 4. Representative Use Case: Energy-Efficient Localization

Localization is a representative sensing-intensive task because it can exploit heterogeneous sensing modalities, such as cellular measurements, Wi-Fi scans, BLE scans, and onboard sensors. As shown in Figure 2, the localization case study follows the four functional components of the TA-MSC framework. Multi-modal sensing data collected from cellular, Wi-Fi, BLE, and onboard sensors are delivered through the network layer to the task processor, where the localization estimator generates the reference-point estimate and confidence-related task-level outputs. These outputs are then used by the task-aware sensing controller to assess whether the current sensing configuration is sufficient and to select the sensing configuration for the next sensing cycle.

In this use case, task sufficiency is evaluated using a confidence-related output of the localization estimator. The controller uses this output to determine whether a candidate sensing configuration is expected to satisfy the required localization condition. It can therefore suppress unnecessary sensing operations when a lower-cost configuration remains sufficient, while richer sensing configurations can be selected when the confidence requirement is not satisfied. The following subsections instantiate the generic TA-MSC objective for localization and describe the modality-wise control actions used to construct feasible sensing configurations.

### 4.1. Task-Constrained Sensing-Control Objective

The localization use case instantiates the generic TA-MSC objective in Equation (1). In this use case, a sensing configuration *c* is represented by a combination of modality-wise action levels, i.e., $c=\{a_{\mathrm{cell}},a_{\mathrm{WiFi}},a_{\mathrm{BLE}},a_{\mathrm{sens}}\}$, where $a_{\mathrm{cell}}$, $a_{\mathrm{WiFi}}$, $a_{\mathrm{BLE}}$, and $a_{\mathrm{sens}}$ denote the selected action levels for cellular reporting, Wi-Fi scanning, BLE scanning, and onboard-sensor activation, respectively. Given this configuration, the sensing-cost term in the generic objective in Equation (1) can be instantiated as the sum of modality-wise energy costs associated with the selected action levels, as follows:

(2) $$J_{c}\left(x\right)=E_{a_{\mathrm{WiFi}}}^{\mathrm{WiFi}}+E_{a_{\mathrm{BLE}}}^{\mathrm{BLE}}+E_{a_{\mathrm{sens}}}^{\mathrm{sens}}+E_{a_{\mathrm{cell}}}^{\mathrm{cell}},$$

where $E_{a_{\mathrm{cell}}}^{\mathrm{cell}},E_{a_{\mathrm{WiFi}}}^{\mathrm{WiFi}},E_{a_{\mathrm{BLE}}}^{\mathrm{BLE}}$, and $E_{a_{\mathrm{sens}}}^{\mathrm{sens}}$ denote the energy costs of the selected cellular, Wi-Fi, BLE, and onboard-sensor action levels, respectively. Specifically, $E_{a_{\mathrm{WiFi}}}^{\mathrm{WiFi}},E_{a_{\mathrm{BLE}}}^{\mathrm{BLE}}$, and $E_{a_{\mathrm{sens}}}^{\mathrm{sens}}$ denote the action-dependent acquisition-energy costs of Wi-Fi, BLE, and onboard sensing, respectively, while $E_{a_{\mathrm{cell}}}^{\mathrm{cell}}$ represents the LTE transmission energy associated with the retained multi-modal payload. The acquisition energy of cellular measurements is not separately modeled because the cellular measurements are treated as already available from the cellular modem in this case study. Instead, the cellular action level changes the amount of retained cellular information and therefore affects the total transmission payload. All retained measurements from Wi-Fi, BLE, cellular, and onboard sensors are assumed to be transmitted through the LTE connection. The payload-dependent LTE transmission energy is modeled using a coefficient of $6.53063333\times 10^{-7}$ J/bit [15]. For the total modeled energy reported in the performance evaluation, the LTE communication term additionally includes a fixed promotion/tail energy of 12.58517 J per request [15]. Because this fixed component is independent of the selected sensing configuration, it does not affect the relative action-dependent sensing decision.

The modality-wise energy parameters are derived from prior measurement studies [15,16,17,18] and converted to a common joule-per-request basis. Table 2 summarizes the original literature-derived parameters and units, the conversion assumptions adopted in this case study, and the resulting modeled energy values. For Wi-Fi, the model uses 1.01 J per channel-equivalent [16], with the resulting per-request energy determined by the number of channel-equivalents associated with each sensing level. For BLE, the reported power of 0.28982 W [17] is converted using a 22.5 s full-scan duration and the active fraction associated with each sensing level.

For onboard sensing, [18] measures each physical sensor at 50 Hz and compares the whole-device power with a dummy sensor that generates software sampling events at the same rate without activating additional sensor hardware. The dummy-sensor power is 573 mW, while the incremental powers of the accelerometer, barometer, light sensor, magnetometer, and gyroscope are 5,1,3,12, and 30 mW, respectively. Since the OutFin sensor measurements used in this case study are sampled at 1 Hz, and each request contains 70 sensor samples, we apply a case-study sampling-rate scaling factor of 1/50 over a 70 s request. Accordingly, the modeled system-and-sampling baseline is calculated as 0.573×(1/50)×70=0.8022 J/request. Adding the corresponding sensor-hardware contributions results in total modeled onboard-sensing energies of 0.8148, 0.8316, and 0.8736 J/request for L1–L3, respectively.

LTE transmission energy is calculated from the retained payload size using $6.530633333\times 10^{-7}$ J/bit [15]. For the total modeled communication energy reported in the performance evaluation, the LTE term additionally includes the 12.58517 J/request promotion/tail energy reported in [15]. These conversions are literature-based modeling assumptions for the present case study and should not be interpreted as direct energy measurements on the OutFin devices.

The estimated task-level performance indicator ${\hat{Q}}_{c}\left(x\right)$ is instantiated as the confidence gap between the top-one and top-two reference-point candidates under sensing configuration *c*. Let $P_{\mathrm{top}1,c(x)}$ and $P_{\mathrm{top}2,c(x)}$ denote the estimated output probabilities of the most likely and second-most likely reference-point candidates when configuration *c* is selected under context *x*, respectively. Then, ${\hat{Q}}_{c}\left(x\right)$ is defined as the difference between these two probabilities, i.e., ${\hat{Q}}_{c}\left(x\right)=P_{\mathrm{top}1,c(x)}-P_{\mathrm{top}2,c(x)}$. Accordingly, the task-level constraint in Equation (1) is instantiated as ${\hat{Q}}_{c}\left(x\right)\geq \Gamma_{\mathrm{loc}}$, where $\Gamma_{\mathrm{loc}}$ denotes the required confidence-gap threshold for localization.

Because the localization task is formulated as reference-point classification, actual localization correctness is evaluated using Top-1 localization accuracy, defined as the fraction of evaluated samples for which the predicted RP matches the ground-truth RP. In contrast, the confidence-satisfaction rate (CSR) is defined as the fraction of evaluated samples for which the selected sensing configuration satisfies the confidence-gap requirement ${\hat{Q}}_{c}\left(x\right)\geq \Gamma_{\mathrm{loc}}$. Therefore, Top-1 accuracy measures actual localization correctness, whereas CSR measures satisfaction of the confidence-based task condition used for task-sufficiency assessment in TA-MSC. The two metrics are reported separately and should not be interpreted as equivalent.

Accordingly, the controller aims to select a low-energy sensing configuration that is expected to satisfy the localization confidence requirement. This differs from unconditional energy minimization because a low-energy configuration is not acceptable if it violates the localization requirement. It also differs from an all-maximum configuration, which activates all available modalities with their richest sensing levels, because such exhaustive sensing is unnecessary when a reduced configuration already provides sufficient confidence. Thus, the objective defines a task-conditioned sensing decision rather than a uniform sensing-reduction rule.

### 4.2. Modality-Wise Control Dimensions

Table 3 summarizes the modality-wise sensing levels used in the localization case study, together with their numerical settings and modeled action-dependent energy costs. Wi-Fi sensing is controlled by scan-channel selection, BLE sensing by the number of retained scan epochs, cellular sensing by the amount of retained serving- and neighbor-cell information, and onboard sensing by sensor-subset activation. Based on these modality-wise sensing levels, the complete Cartesian action space contains 5×5×4×4=400 candidate sensing configurations. Each action specifies an absolute target sensing configuration consisting of one Wi-Fi, BLE, cellular, and onboard-sensor level.

### 4.3. Case-Study Evaluation

We evaluate the localization use case using the OutFin dataset [1], which contains multi-modal measurements collected at 122 reference points, including Wi-Fi AP scans, BLE beacon measurements, cellular information, and onboard sensor readings. The localization task is formulated as reference-point classification. In this offline localization case study, the general evaluation mechanism described in Section 3.2 is instantiated by deterministically retaining the recorded measurements corresponding to each candidate sensing configuration. The resulting measurements are then processed by the localization estimator, which uses only the features allowed by the selected modality-wise action levels and outputs a reference-point prediction together with the output probabilities used to compute the confidence gap.

The localization estimator is implemented as a deterministic 122-class prototype-based classifier. Phone 1 measurements are used to construct a representative prototype for each of the 122 reference points (RPs). The input features depend on the sensing modality. Wi-Fi and BLE use the detected access-point or beacon identifiers together with their RSS measurements. Cellular input consists of the measurement fields retained by the selected cellular level in Table 3, while onboard sensing uses the sensor measurements retained by the corresponding sensor level. Repeated measurements are summarized using median values, and numerical measurements are normalized using statistics obtained from Phone 1. For each candidate sensing configuration, only the measurements retained by the corresponding modality-wise action levels are used. The resulting feature vector of each modality is compared with the Phone 1-derived RP prototypes using cosine similarity, from which a probability distribution over the 122 candidate RPs is obtained. The modality-specific probability distributions are combined using reliability weights determined from their prediction entropy, so that a modality producing a more concentrated RP probability distribution contributes more strongly to the fused result. The RP with the highest fused probability is selected as the localization result, and the two highest probabilities are used to calculate the confidence gap. Phone 1 is used to construct the RP prototypes and pre-processing parameters, whereas Phone 2 is evaluated using the fixed Phone 1-derived estimator without Phone 2-specific estimator fitting.

For the case-study evaluation, $\Gamma_{\mathrm{loc}}$ is set to 0.01667, corresponding to the 10th-percentile (q10) confidence-gap value obtained from Phone 1 sensing configurations that preserve the same RP prediction as the corresponding All-Max request. For this case study, q10 is used as a low-end evaluation threshold, whereas a q15 value of 0.05948 is used only as a slightly stricter training guard margin. Since q15 is higher than q10, the training condition encourages the policy to maintain a confidence margin above the final evaluation boundary. Both percentiles are derived exclusively from Phone 1 and fixed before the Phone 2 evaluation. These percentile choices are case-study-specific design parameters rather than universal optima. Accordingly, the resulting energy–confidence tradeoff may vary with the selected confidence operating point.

The sensing controller is implemented using a Lagrangian RL approach [19]. The state is represented as $s=(a_{W},a_{B},a_{C},a_{S},b_{g},b_{H})$, where $a_{W}$, $a_{B}$, $a_{C}$, and $a_{S}$ denote the current Wi-Fi, BLE, cellular, and onboard-sensor levels, respectively, and $b_{g}$ and $b_{H}$ are 11-bin discretizations of the fused confidence gap and prediction entropy. Each action selects one of the 400 absolute sensing configurations defined in Table 3. The reward is defined as $r=-E_{\mathrm{norm}}-\lambda \kappa I_{\mathrm{viol}}$, where $E_{\mathrm{norm}}$ denotes the normalized modeled energy cost, λ is the Lagrange multiplier, κ is the violation-penalty scale, and $I_{\mathrm{viol}}$ is a binary source-domain service-violation indicator. During Phone 1 training, $I_{\mathrm{viol}}=1$ when the selected configuration does not preserve the corresponding All-Max RP prediction or does not satisfy the q15 confidence-margin condition described above. Within each outer training block, the Q policy is updated while λ is held fixed. At the end of each block, λ is updated according to the measured source-domain violation rate relative to the target violation rate. The main implementation parameters and training settings are summarized in Table 4. Two independent RL training runs were conducted using random seeds 11 and 22. A third TA-MSC policy used in the evaluation was constructed by merging the two independently trained policies and therefore does not constitute an additional independent RL run. Accordingly, the reported mean ± standard deviation values for TA-MSC summarize the three evaluated policy instances and are interpreted as descriptive policy-instance variability rather than variability across three independent RL training runs.

For comparison, we use the All-Max configuration as a full-sensing baseline [1], which activates all available modalities at their richest sensing levels. We additionally evaluate a STATIC baseline that uses the fixed sensing configuration (L3,L3,L2,L2), corresponding to a one-level reduction from the maximum level of each modality. All four modalities therefore remain active, while the sensing effort is moderately reduced in a simple and consistent manner across modalities. This baseline is not intended to represent an optimized static policy; rather, it serves as a non-adaptive reference for assessing whether TA-MSC provides additional energy savings beyond those obtained by reducing each modality by one discrete level. After the common initial All-Max request used to initialize each evaluation scenario, STATIC applies this fixed configuration to all subsequent controlled requests without using task-level feedback. Figure 3 compares the mean reference-based modeled sensing energy of All-Max, STATIC, and TA-MSC under the same Phone 2 evaluation scenarios. The corresponding values are 58.386 J, 40.547 J, and 27.578±3.859 J, respectively. Thus, TA-MSC reduces the modeled sensing energy by 52.77±6.61% relative to All-Max and by 31.99±9.52% relative to STATIC. The modality-wise decomposition in Figure 3 further shows that the energy reduction is not uniformly distributed across sensing modalities. In particular, Wi-Fi accounts for a substantial portion of the reduction relative to All-Max, whereas the other modalities are retained at relatively higher sensing levels when required by the task-level feedback. This result indicates that TA-MSC does not simply reduce all sensing levels uniformly; instead, it adaptively adjusts the modality-wise sensing configuration according to the current task condition.

Figure 4 further examines the reference-point-wise and modality-wise modeled-energy saving ratios of TA-MSC using two reference configurations. Figure 4a shows the saving ratio relative to All-Max, demonstrating that the available energy-saving opportunity varies considerably across reference points and sensing modalities rather than being uniform across the evaluated scenarios. At reference points where TA-MSC retains a sensing configuration close to All-Max, the resulting energy saving becomes small or nearly zero. Larger savings are obtained when lower sensing levels can be selected based on the current task-level state. Figure 4b shows the saving ratio relative to STATIC. Although TA-MSC achieves additional energy reduction over STATIC for many reference-point/modality combinations, some entries exhibit negative savings, indicating that TA-MSC uses more sensing energy than the fixed STATIC configuration in those cases. This behavior reflects the adaptive nature of the proposed policy: TA-MSC does not always reduce sensing effort, but can retain richer sensing levels when indicated by the current task-level state. Taken together, the two comparisons demonstrate that TA-MSC performs context- and modality-dependent sensing adaptation rather than applying a uniform sensing-reduction rule.

Figure 5 further compares the Top-1 localization accuracy and CSR of the three schemes under the same Phone 2 evaluation scenarios. The Top-1 accuracies are 0.9737 for All-Max, 0.9540 for STATIC, and 0.9614±0.0095 for TA-MSC, while the corresponding CSRs are 0.9768, 0.9780, and 0.9894±0.0039, respectively. These results demonstrate that confidence-constraint satisfaction and actual localization correctness represent different aspects of task performance. TA-MSC achieves the highest CSR, indicating that its selected sensing configurations most frequently satisfy the predefined confidence-gap requirement, whereas All-Max achieves the highest Top-1 localization accuracy. Accordingly, the confidence gap in this case study is interpreted as a task-sufficiency indicator for sensing control rather than as a direct measure of localization correctness.

## 5. Limitations and Open Research Issues

The present study has several limitations. First, the case study is based on the offline OutFin dataset rather than a real-time sensing platform. Second, modality-wise sensing energy is modeled using values derived from prior measurement studies rather than directly measured on the evaluated devices. Third, the top-one/top-two confidence gap is used as a surrogate task-level indicator and may not always coincide with actual localization correctness. In addition, the selected confidence operating point is case-study-specific, and different confidence thresholds may lead to different tradeoffs among modeled sensing energy, localization accuracy, and confidence satisfaction. Finally, the complete TA-MSC closed loop has not yet been validated on a physical multi-modal IoT platform. These limitations define the scope of the present evaluation and motivate further investigation of the broader research issues discussed below.

### 5.1. Reliable Task-Level Output Modeling

In online operation, ground-truth labels are often unavailable, which makes it difficult to directly compute task errors. Therefore, the controller may need to rely on surrogate indicators produced by the inference process, such as confidence or uncertainty. However, surrogate task-level outputs do not always accurately reflect actual task performance. For example, a learning-based localization model may assign a high confidence score to an incorrect reference-point prediction when the sensing data are noisy, the environment changes, or the deployment condition differs from the training data. More generally, uncertainty arising from sensing and inference can affect localization reliability, and uncertainty-aware methods have been used to quantify the reliability of multi-modal localization outputs [20]. In the TA-MSC setting specifically, whether a sensing configuration satisfies the required task condition can be affected by measurement noise, modality availability, environmental variation, and channel conditions. For RF-based sensing modalities, physical-layer quality indicators, such as signal-to-noise ratio (SNR), may therefore be incorporated as modality-specific context when estimating task sufficiency. However, because heterogeneous sensing modalities exhibit different sensing and noise characteristics, a single SNR-based metric may not be universally applicable; different modalities may require modality-specific quality or uncertainty indicators. Therefore, calibrated and robust task-level indicators should be developed so that task-sufficiency assessment remains reliable across different sensing and deployment conditions.

### 5.2. Stable and Practical Sensing-Control Policies

TA-MSC can be implemented using rule-based control, optimization-based methods, reinforcement learning, or hybrid approaches. Each method has different advantages in terms of simplicity, adaptability, optimality, and implementation cost. However, no single control approach is suitable for all task requirements, device capabilities, and deployment conditions. Therefore, practical sensing-control policies should be designed to balance stability, responsiveness, and sensing efficiency while preventing frequent changes of sensing configurations caused by noisy or delayed task-level outputs.

### 5.3. Interoperability Between Functional Components

Practical TA-MSC operation requires interoperability among sensing sources, the task processor, and the task-aware sensing controller. The controller should recognize available modalities, sensing costs, measurement availability, and supported control dimensions, while sensing sources should understand and execute control decisions. However, current sensing platforms do not usually expose such control-relevant information through unified interfaces, and multi-modal data may be collected asynchronously due to different sampling periods and reporting delays. Therefore, lightweight interfaces and data-alignment mechanisms should be designed to support controller-readable sensing information and task-level outputs.

### 5.4. Deployment of Task Processor and Sensing Controller

The task processor and sensing controller can be deployed on the device, at the edge, or in the cloud. Each deployment option has different tradeoffs in terms of feedback latency, computation capability, energy consumption, communication overhead, and privacy. However, current IoT systems do not provide clear guidelines for determining where task processing, task-sufficiency assessment, and sensing-control decisions should be performed. Therefore, deployment architectures and functional split strategies should be investigated to adapt TA-MSC operation to device capability, network condition, task latency, and privacy requirements.

## 6. Conclusions

Future IoT systems require sensing operations that can adapt sensing effort according to task-level requirements under limited energy and communication resources. This article presented Task-Aware Multi-Modal Sensing Control (TA-MSC), a task-level-output-driven closed-loop framework that seeks low-cost sensing configurations while satisfying an explicit task-level performance requirement. Rather than treating sensing quality only at the signal or sensor level, TA-MSC connects task-level inference outputs directly to sensing-control decisions and jointly adjusts heterogeneous modality-specific sensing levels through practical control dimensions such as modality selection, sensor activation, channel adjustment, and duty-cycle management. In the evaluated cross-device localization case study, TA-MSC reduces the mean total modeled sensing energy by 52.8% relative to All-Max. All-Max achieves the highest Top-1 localization accuracy at 97.37%, whereas TA-MSC achieves 96.14%; in contrast, TA-MSC achieves a confidence-satisfaction rate of 98.94%, compared with 97.68% for All-Max. These results show that TA-MSC provides substantial modeled-energy savings with a modest reduction in Top-1 localization accuracy, while more frequently satisfying the confidence-based task condition. At the same time, the current evaluation is limited to a localization-oriented case study and modeled sensing costs, and broader validation is still required across different tasks, sensing platforms, and dynamic operating conditions. In our future work, we will implement TA-MSC on a real multi-modal IoT platform and evaluate its behavior under practical device, network, and workload variations, while also extending the framework to other task-level outputs and sensing applications.

## Figures and Tables

**Figure 1:**
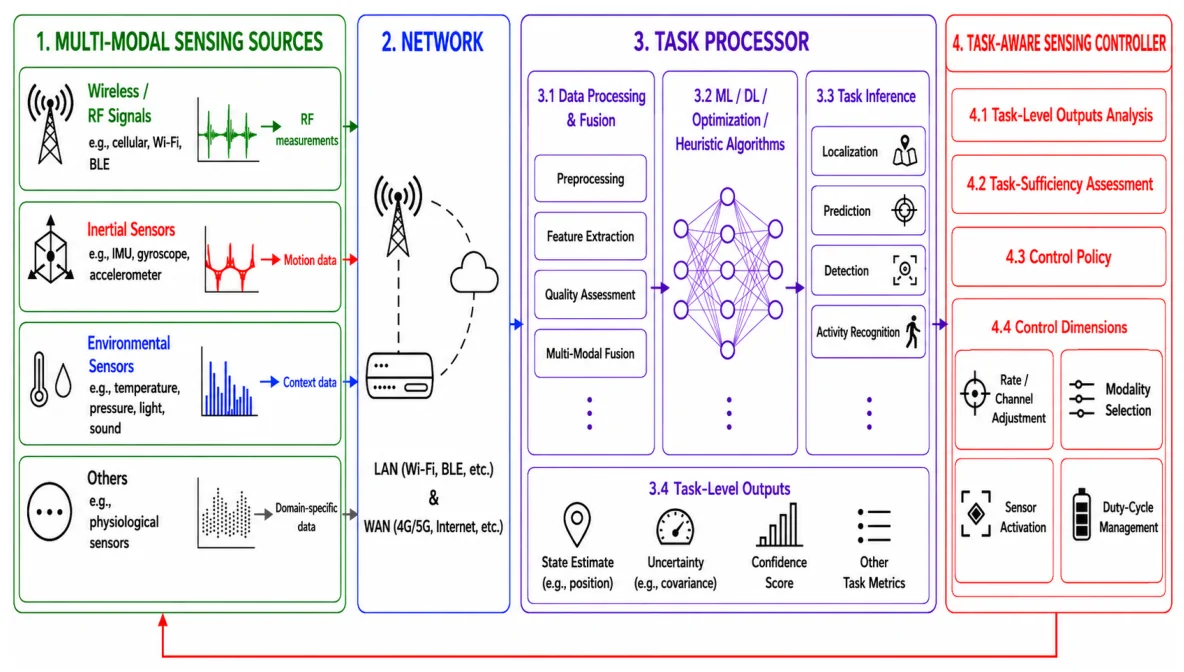
Conceptual architecture and closed-loop information/control flow of TA-MSC. The sensing sources and communication technologies shown are representative examples rather than an exhaustive set.

**Figure 2:**
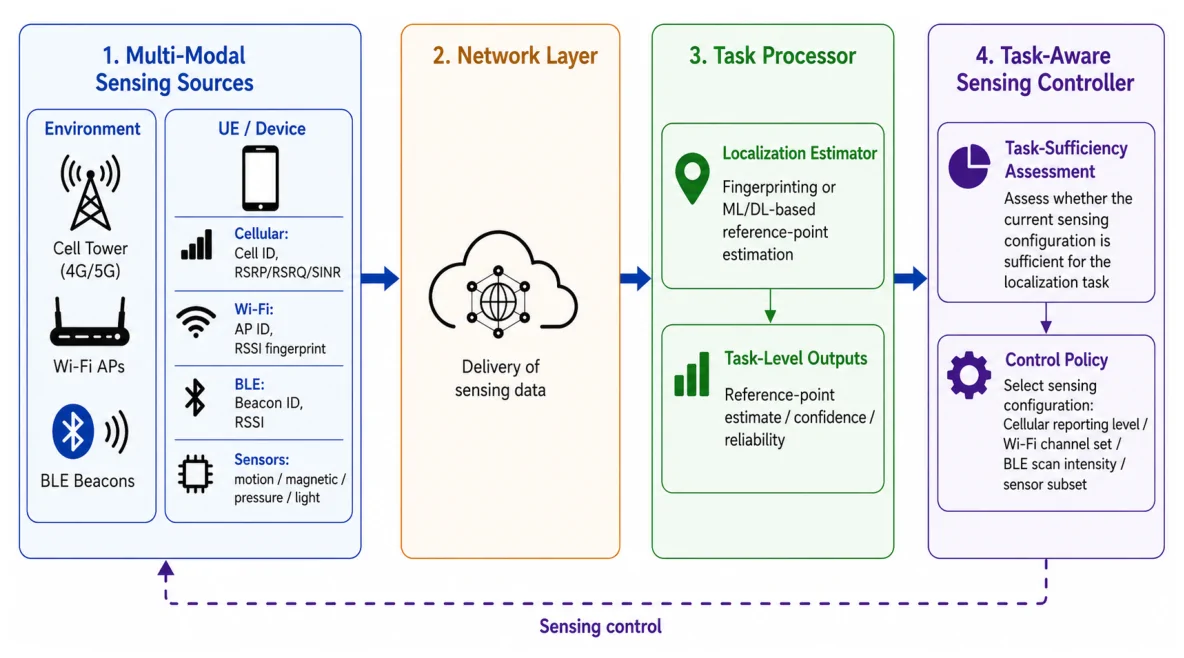
Energy-efficient localization use case.

**Figure 3:**
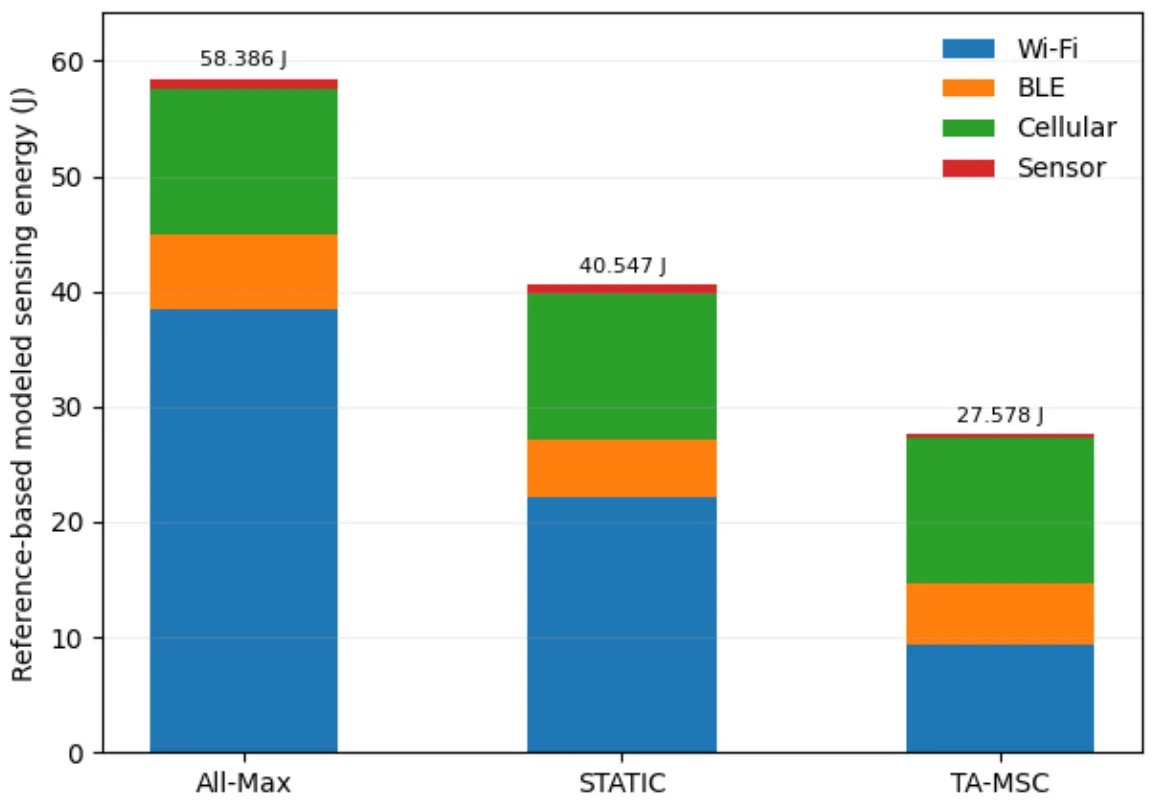
Average modality-wise modeled sensing energy of the all-maximum configuration, STATIC, and the proposed TA-MSC policy.

**Figure 4:**
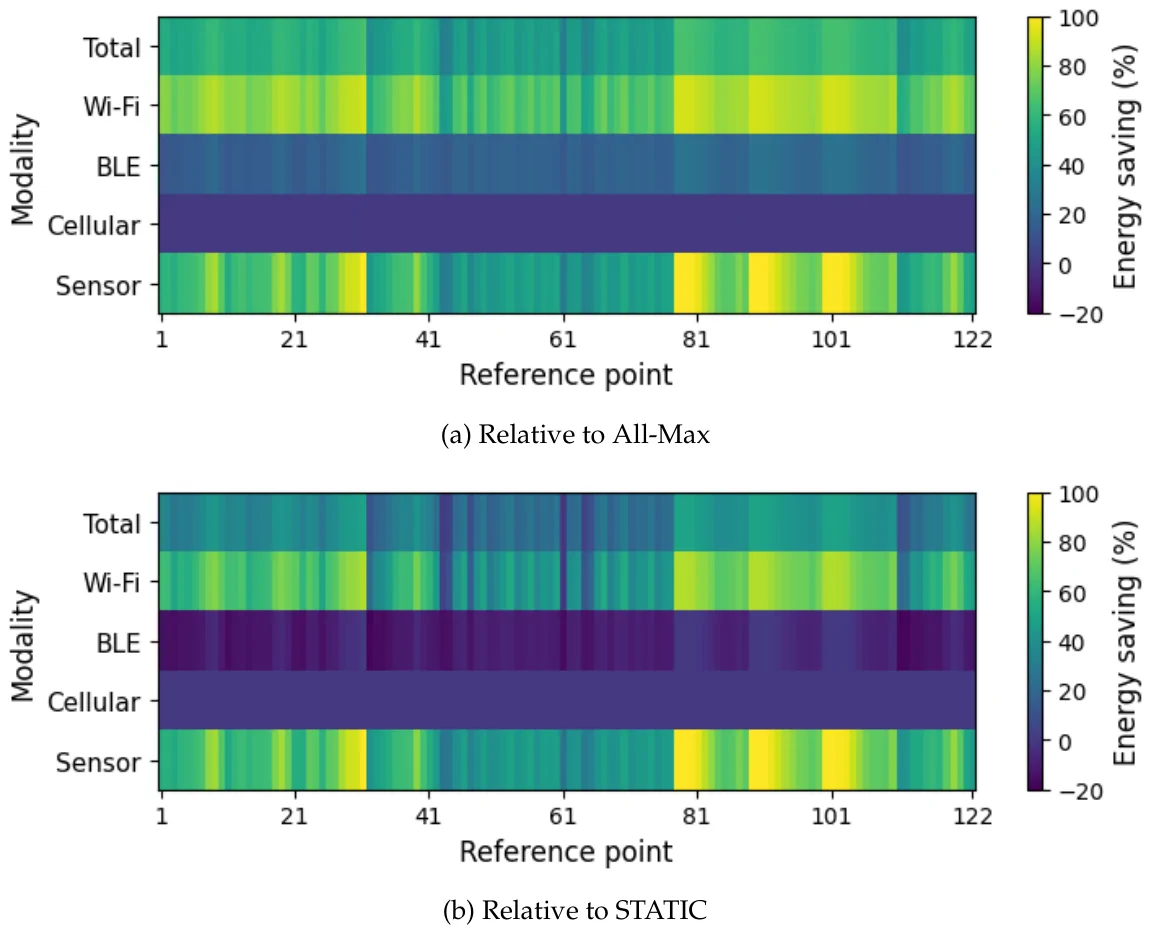
Reference-point-wise and modality-wise modeled-energy saving ratios of the proposed TA-MSC policy relative to (**a**) All-Max and (**b**) STATIC under the Phone 2 evaluation scenarios.

**Figure 5:**
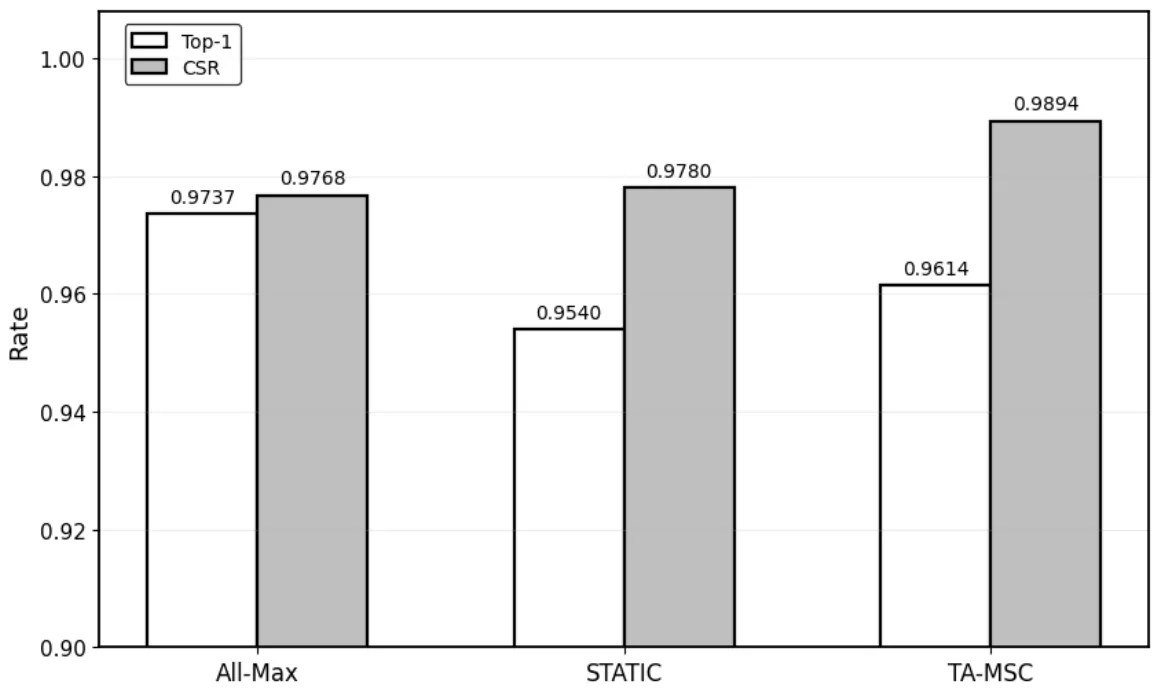
Top-1 localization accuracy and CSR of All-Max, STATIC, and TA-MSC under the Phone 2 evaluation scenarios.

**Table 1:** Comparison of representative adaptive, task-aware, and multi-modal sensing approaches.

Work	Application	Multi-Modal Inference	Decision Basis	Sensing Control	Task-Performance Treatment
Murad et al. [8]	Environmental sensing	No	Prediction uncertainty and sensing information	Measurement timing and sparse sensing	Model uncertainty is used to determine when additional measurements are informative and sensing resources should be allocated.
CM-ASAP [9]	Multi-modal perception	Yes	Task prediction and expected accuracy benefit	Modality selection and sensing-rate adaptation	Task-related benefit of additional sensing is used to adapt multi-modal sensing configurations.
ReSPEC [10]	Multi-modal perception	Yes	Modality-specific task information and system context	Sampling frequency, resolution, and sensing range	Task-related modality information is incorporated directly into learning-based sensing reconfiguration.
Zhou et al. [11]	Fingerprint localization	No	Mutual information and expected localization accuracy	Sampling interval and sample capacity	Expected localization accuracy is used to derive the minimum sample capacity required for fingerprint-database construction.
Shurrab et al. [12]	Target localization	No	Sensor readings and node attributes	Search-region selection and active sensor selection	RL guides the localization process, while sensor attributes such as activation cost, residual energy, confidence, and distance are considered for node selection.
Ghosh et al. [13]	Multi-sensing WSN	No	Inter-sensor correlation, sensing cost, and available energy	Active sensor-subset selection	Sensor selection is primarily guided by sensing-side relationships and resource conditions, while measurements of inactive sensors are estimated.
AMSER [14]	Multi-modal eHealth sensing	Yes	Input signal quality and cross-modal discrepancy	Feature/modality selection and sensing reconfiguration	Multi-modal sensing is adapted according to input quality to improve inference robustness while reducing sensing energy.
**TA-MSC**	**Multi-modal localization**	**Yes**	**Task-level inference output and sensing cost**	**Joint heterogeneous multi-level sensing control**	**The sensing configuration is selected to reduce sensing cost while satisfying an explicit task-level performance requirement evaluated from inference outputs.**

**Table 2:** Traceability of literature-derived energy parameters and their conversion to the modeled per-request energy used in the localization case study.

Component	Literature-Derived Parameter	Case-Study Conversion	Resulting Modeled Energy
Wi-Fi [16]	1.01 J/channel-equivalent	$1.01\times n_{\mathrm{ch}}$, where $n_{\mathrm{ch}}=\left\{0,3,13,22,38\right\}$ for L0–L4	0, 3.03000, 13.13000, 22.22000, and 38.38000 J/request
BLE [17]	0.28982 W	0.28982×22.5×f, where f={0,0.25,0.50,0.75,1.00} for L0–L4	0, 1.63024, 3.26048, 4.89071, and 6.52095 J/request
Onboard sensors [18]	Dummy: 573 mW; incremental power: accelerometer 5 mW, barometer 1 mW, light 3 mW, magnetometer 12 mW, and gyroscope 30 mW; measured at 50 Hz	P×(1/50)×70 s. The dummy baseline is 0.573×(1/50)×70=0.8022 J/request	Hardware contribution for L1–L3: 0.01260, 0.02940, and 0.07140 J/request; total modeled energy: 0.8148, 0.8316, and 0.8736 J/request
LTE transmission [15]	$6.530633333\times 10^{-7}$ J/bit; promotion/tail: 12.58517 J/request	$E_{\mathrm{Tx}}=6.530633333\times 10^{-7}N_{\mathrm{bit}}$	Payload-dependent transmission energy; total reported communication energy additionally includes 12.58517 J/request

**Table 3:** Modality-wise sensing actions and modeled action-dependent energy costs used in the localization case study.

Modality	Level	Sensing Configuration	Numerical Setting/Retained Information	Energy (J/request)
Wi-Fi	L0	Off	No Wi-Fi measurements retained	0
	L1	Selected 2.4 GHz channels	Channels 1, 6, and 11	3.03000
	L2	Full recorded 2.4 GHz scan	All recorded 2.4 GHz channels	13.13000
	L3	2.4 GHz + selected 5 GHz channels	All recorded 2.4 GHz channels + non-DFS 5 GHz channels 36, 40, 44, 48, 149, 153, 157, 161, and 165	22.22000
	L4	Full recorded Wi-Fi scan	All recorded 2.4/5 GHz Wi-Fi measurements	38.38000
BLE	L0	Off	No BLE scan epochs retained	0
	L1	Reduced scan	First 2 recorded scan epochs	1.63024
	L2	Reduced scan	First 4 recorded scan epochs	3.26048
	L3	Reduced scan	First 6 recorded scan epochs	4.89071
	L4	Full scan	All recorded scan epochs	6.52095
Cellular	L0	Basic serving-cell information	ECI + RSRP	–^*^
	L1	Extended serving-cell information	L0 + EARFCN + RSRQ	–^*^
	L2	Serving- and neighbor-cell information	L1 + LTE_neighbors + RSRP_strongest	–^*^
	L3	Full cellular information	L2 + timing advance (TA)	–^*^
Onboard sensors	L0	Off	No onboard-sensor measurements retained	0
	L1	Basic sensor set	Accelerometer + pressure + illuminance	0.8148
	L2	Extended sensor set	L1 + magnetometer	0.8316
	L3	Full sensor set	L2 + gyroscope	0.8736

* The acquisition energy of cellular measurements is not separately modeled because cellular measurements are treated as already available from the cellular modem. Cellular action levels instead change the retained information and thereby affect the LTE transmission payload.

**Table 4:** Implementation settings of the Lagrangian RL controller.

Item	Setting
State	$\left(a_{W},a_{B},a_{C},a_{S},b_{g},b_{H}\right)$; four current sensing levels, 11 confidence-gap bins, and 11 entropy bins
Action	5×5×4×4=400 absolute sensing configurations
Reward	$r=-E_{\mathrm{norm}}-\lambda \kappa I_{\mathrm{viol}}$
Violation condition	All-Max RP prediction is not preserved, or the q15 training-margin condition is not satisfied
Discount factor γ	0.90
Base Q-learning rate α	0.025
Penalty scale κ	300
Target violation rate	0.001
Lagrange multiplier	$\lambda_{\mathrm{init}}=0.05$, $\lambda_{\max}=25$
Dual step size	$\beta_{k}=0.02/\left[1+\left(k-1\right)/50\right]$
Training iterations	80 outer blocks × 70 primal epochs
Training per epoch	10 training scenarios and 600 replay TD updates
Exploration	ϵ:0.04→0.0005
Independent training seeds	11 and 22
Additional evaluation policy	Merged policy derived from the two independently trained policies

## Data Availability

The dataset analyzed in this study is publicly available in the OutFin repository on Figshare at https://doi.org/10.6084/m9.figshare.13370291 (accessed on 12 September 2026). The results reported in this study were generated by the authors based on this publicly available dataset.
